# Plastid genome of *Chenopodium petiolare* from Trujillo, Peru

**DOI:** 10.1186/s13104-024-06705-y

**Published:** 2024-03-11

**Authors:** Flavio Aliaga, Mario Zapata-Cruz, Silvia Ana Valverde-Zavaleta

**Affiliations:** 1 Grupo de Investigación en Ecología Evolutiva, Protección de Cultivos, Remediación Ambiental, y Biotecnología (EPROBIO), Universidad Privada del Norte, Trujillo, 13011 Peru; 2 Dirección de Investigación, Innovación y Responsabilidad Social, Universidad Privada del Norte, Trujillo, 13009 Peru; 3 Capítulo de Ingeniería Agronómica, Consejo Departamental de La Libertad (CDLL), Colegio de Ingenieros del Perú (CIP), Trujillo, 13008 Peru; 4 Plant Science Laboratory (PSL), Trujillo, 13009 Peru

**Keywords:** Plastid genome, Chloroplast genome, Chenopodiaceae, *Chenopodium petiolare*, Lomas del Cerro Campana, Trujillo, Peru

## Abstract

**Objectives:**

The Peruvian Andean region is an important center for plant domestication. However, to date, there have been few genetic studies on native grain, which limits our understanding of their genetic diversity and the development of new genetic studies for their breeding. Herein, we revealed the plastid genome of *Chenopodium petiolare* to expand our knowledge of its molecular markers, evolutionary studies, and conservation genetics.

**Data description:**

Total genomic DNA was extracted from fresh leaves (voucher: USM < PER > :MHN333570). The DNA was sequenced using Illumina Novaseq 6000 (Macrogen Inc., Seoul, Republic of Korea) and reads 152,064 bp in length, with a large single-copy region of 83,520 bp and small single-copy region of 18,108 bp were obtained. These reads were separated by a pair of inverted repeat regions (IR) of 25,218 bp, and the overall guanine and cytosine (GC) was 37.24%. The plastid genome contains 130 genes (111 genes were unique and 19 genes were found duplicated in each IR region), including 86 protein-coding genes, 36 transfer RNA-coding genes, eight ribosomal RNA-coding genes, and 25 genes with introns (21 genes with one intron and four genes with two introns). The phylogenetic tree reconstructed based on single-copy orthologous genes and maximum likelihood analysis indicated that *Chenopodium petiolare* is most closely related to *Chenopodium quinoa*.

## Objective

*Chenopodium petiolare* Kunth is a native grain of the Andean region, this annual herb grows in the Peruvian Andean formations at altitudes of 200–3,900 m.a.s.l., and its grains are small and black with high concentration of saponins [1, 2]. It is a diploid species with a small number of chromosomes (2n = 2x = 18) belonging to the Chenopodiaceae family. Its outstanding features are drought stress tolerance and resistance to diseases [1, 3]. *Chenopodium petiolare* has multiple uses including being used as cattle feed, in cooking local dishes such as quispiño (dark muffin), and in traditional medicine mainly for bone fractures [1].

The plastid genome has a quadripartite structure: a large single-copy (LSC) of 80–90 kilobase pairs (kb), a small single-copy (SSC) of 16–27 kb, and two sets of inverted repeats (IRs) of 20–28 kb, with 110–130 unique genes, including protein-coding genes, transfer RNA (tRNA), and ribosomal RNA (rRNA) [4, 5]. In recent years, declining genome sequencing costs resulted in more than 790 complete plant genomes of different species becoming available [6, 7]. Recently, some *Chenopodium* plastid genomes such as *Chenopodium acuminatum* [8], *Chenopodium album* [9], *Chenopodium quinoa* [10], *Chenopodium ficifolium* [11], became publicly available. However, despite the few genetic data available, we have only begun to investigate the genomics of native grains of great importance for plant breeding programs. In the present study, we report the first plastid genome sequence submitted for an isolate of *Chenopodium petiolare*, which will expand our knowledge about its plant molecular breeding, molecular markers, evolutionary studies, and conservation genetics.

## Data description

**Fig. 1:**
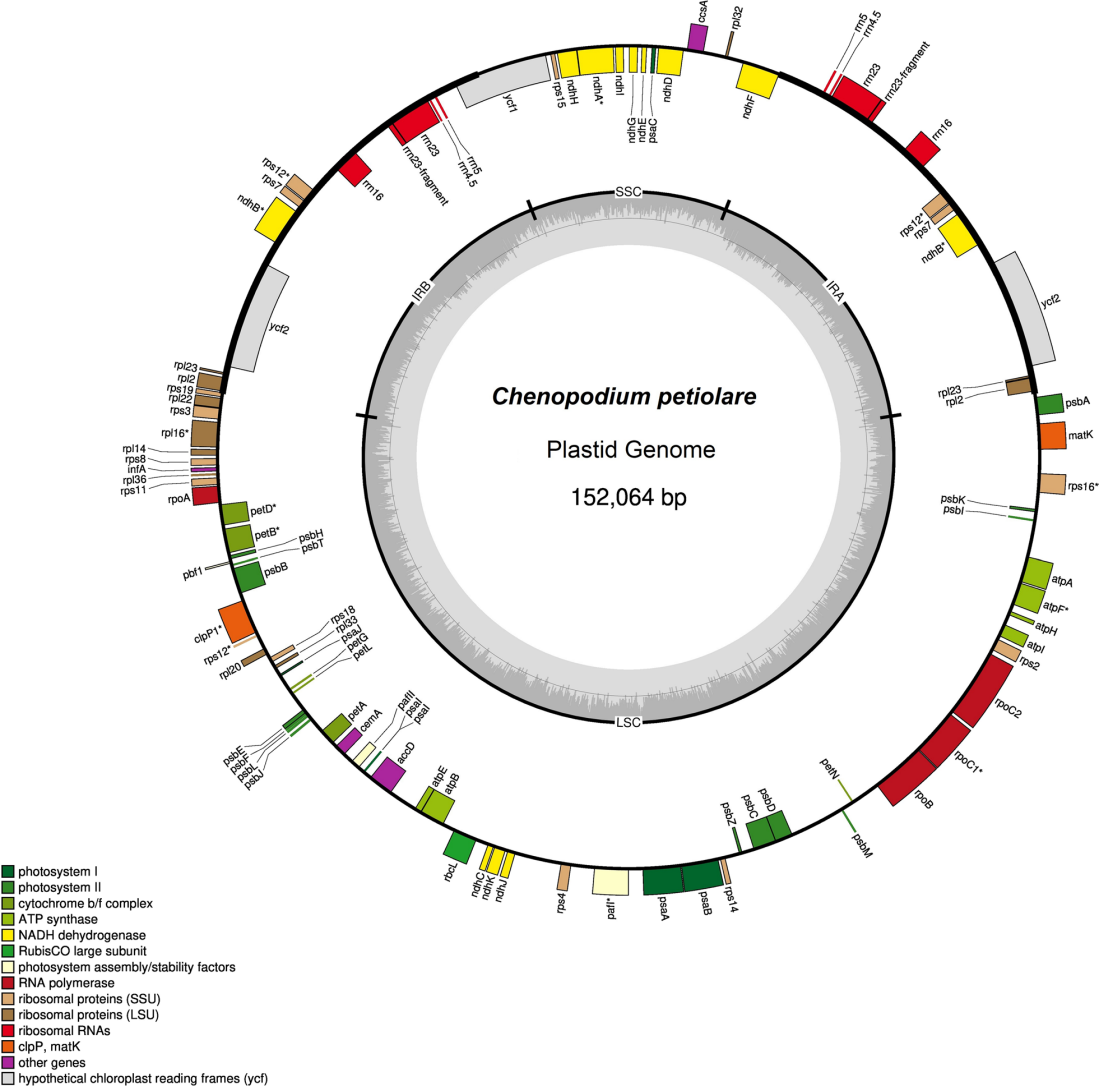
Circular map of *Chenopodium petiolare* chloroplast genome. The thick lines indicate the IR1 and IR2 regions, which separate the large single-copy (LSC) and small single-copy (SSC) regions. Genes marked inside the circle are transcribed clockwise, and genes marked outside the circle are transcribed counterclockwise. Genes are color-coded based on their function, shown at the bottom left. The inner circle indicates the inverted boundaries and guanine and cytosine (GC) content

Total genomic DNA was extracted from approximately 100 mg of fresh leaves (from voucher number USM < PER > :MHN333570) (Data file 1) using a cetyl-trimethyl ammonium bromide (CTAB) protocol [12]. Genomic DNA quality was assessed using a fluorometry-based Qubit (Thermo Fisher Scientific, USA) coupled to a Broad Range Assay kit (Thermo Fisher Scientific, USA). High-quality DNA (230/260 and 260/280 ratios > 1.8) was normalized (20 ng/μL) to examine its integrity using 1% (*w*/*v*) agarose gel electrophoresis. Qualified DNA was fragmented, and the TruSeq Nano DNA kit (Illumina, San Diego, CA, USA) was used to construct an Illumina paired-end (PE) library. PE sequencing (2 × 150 bp) was performed using the Illumina NovaSeq 6000 platform (Macrogen, Inc., Seoul, Republic of Korea) [13]. All adapters and low-quality reads were removed using the FastQC [14] and Cutadapt [15] programs. PE reads (2 × 150 bp) were evaluated for quality using QUAST [16] analysis, and subsequent steps used clean data. Then, clean reads obtained were assembled into a circular contig using NOVOPlasty (version.4.3) [17], with *C. quinoa* (NC_034949) as the reference. Data can be accessed from NCBI GenBank under the accession number OQ957163 [30]. The plastid genome was annotated using the Dual Organellar GenoMe Annotator GeSeq [18] and CpGAVAS2 [19]. A circular genome map was constructed using OGDRAW (version 1.3.1) [20] (Fig. 1). The plastid genome encoded 130 genes, of which 111 were unique, and 19 were duplicated in the inverted repeat (IR) region. The chloroplast genome contained 86 protein-coding genes, 36 tRNA-coding genes, eight rRNA-coding genes, and 25 genes with introns (21 genes with one intron and four genes with two introns), as shown in Data file 3.

**Fig. 2:**
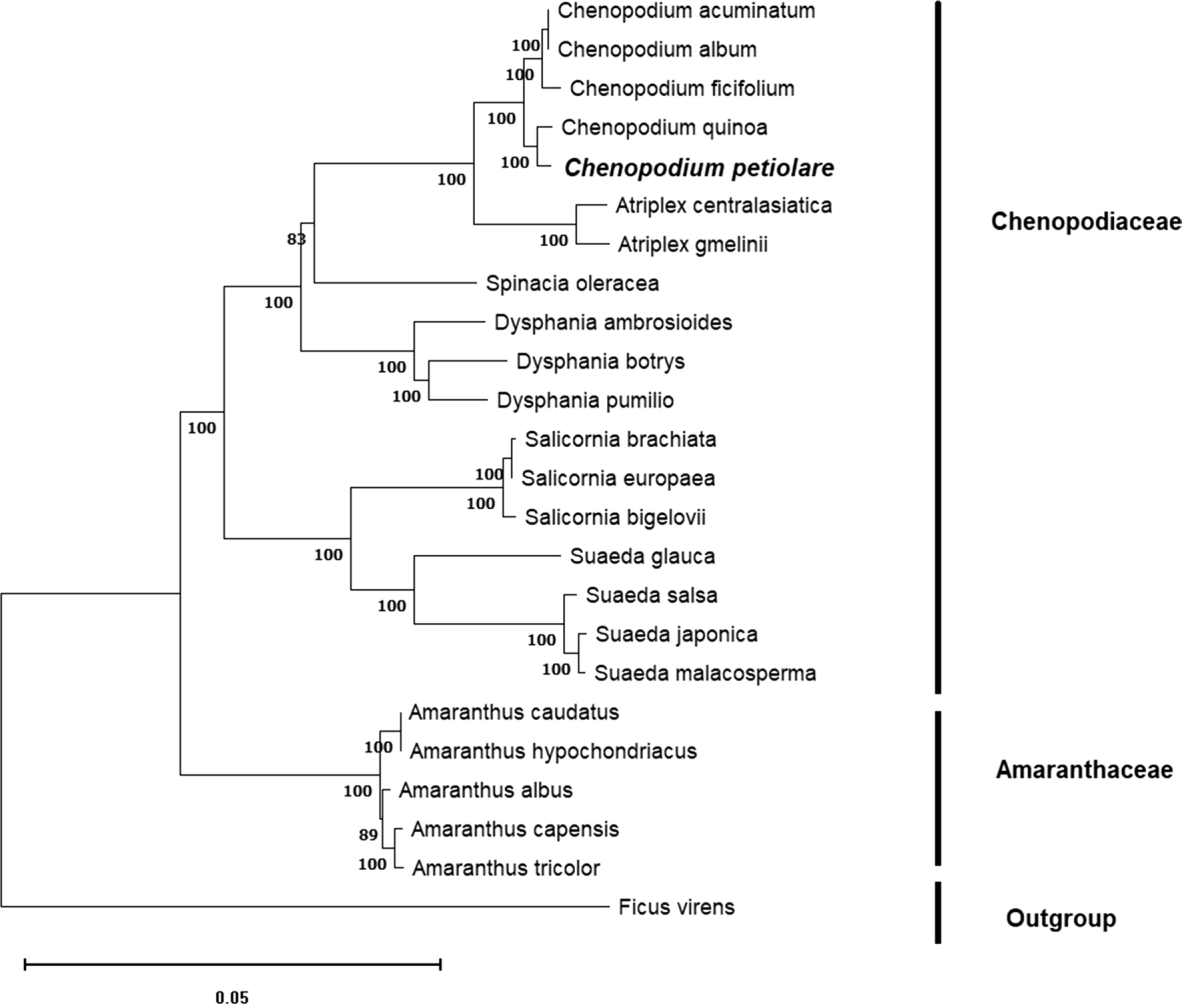
Phylogenetic tree of 24 plastid genomes. Maximum likelihood analysis based on single-copy orthologous protein. Bootstrap values on the branches were calculated from 1000 replicates

The plastome contained 111 unique genes, of which there were 28 tRNA genes, four rRNA genes, and 79 protein-coding genes. The latter comprised 21 ribosomal subunit genes (nine large subunits and 12 small subunit), four DNA-directed RNA polymerase genes, 45 genes were involved in photosynthesis (11 encoded subunits of the NADH oxidoreductase, seven for photosystem I, 14 for photosystem II, six for the cytochrome b6/f complex, six for different subunits of ATP synthase, and one for the large chain of ribulose biphosphate carboxylase), eight genes were involved in different functions, and one gene was of unknown function (Data file 4). Phylogenetic analysis reconstruction was performed using 24 complete chloroplast genome sequences to infer the phylogenetic relationships among *Chenopodium* species, and *Ficus virens* was used as an outgroup (Fig. 2). Single-copy orthologous genes were identified using the Orthofinder pipeline (version 2.2.6) [21]. For each gene family, the nucleotide sequences were aligned using the L-INS-i algorithm in MAFFT (version 7.453) [22]. A phylogenetic tree based on maximum likelihood (ML) was constructed using RAxML (version 8.2.12) [23] with the GTRCAT model. A phylogenetic ML tree was reconstructed and edited using MEGA (version 11) [24] with 1000 replicates. The phylogenetic tree illustrated that *Chenopodium petiolare* is closely related to *Chenopodium quinoa* [10].

## Limitations

This study used leaf samples of *Chenopodium petiolare* from the Lomas del Cerro Campana Private Conservation Area in Trujillo, Peru. Administratively, this process takes longer than necessary to obtain the corresponding access permit for plant sample collection.

## Data Availability

The data described in this Data note can be freely and openly accessed on GenBank of NCBI repository under the accession number OQ957163, and figshare. Please see Table

**Table 1:** Overview of data files/data sets

Label	Name of data file/data set	File types (file extension)	Data repository and identifier (DOI or accession number)
*Data file 1*	Herbarium specimen voucher of *Chenopodium petiolare* Kunth (USM < PER > :333,570)	*Picture file (.jpg)*	*Figshare* https://doi.org/10.6084/m9.figshare.23574303.v1 [25]
*Data file 2*	Figure 1 Circular map of *Chenopodium petiolare* plastid genome	*Picture file (.jpg)*	*Figshare* https://doi.org/10.6084/m9.figshare.23574270.v1 [26]
*Data file 3*	Plastid genome features of the* Chenopodium petiolare*	*Document file (.docx)*	*Figshare* https://doi.org/10.6084/m9.figshare.23574306.v1 [27]
*Data file 4*	Genes present in the plastid genome of *Chenopodium petiolare*	*Document file (.docx)*	*Figshare* https://doi.org/10.6084/m9.figshare.23574312.v1 [28]
*Data file 5*	Figure 2 Phylogenetic tree of 24 plastid genomes	*Picture file (.jpg)*	*Figshare* https://doi.org/10.6084/m9.figshare.23574327.v1 [29]
*Data set 1*	*Chenopodium petiolare* chloroplast, complete genome	*Fasta file (.fasta)*	GenBank from NCBI repository under the accession number OQ957163 (https://identifiers.org/ncbi/insdc:OQ957163) [30]

1 and references list [25–30] for details and links to the data. Overview of data files/data sets GenBank from NCBI repository under the accession number OQ957163 (https://identifiers.org/ncbi/insdc:OQ957163) [30]

## Abbreviations

LSC: Large single-copy
SSC: Small single-copy
IR: Inverted repeat
tRNA: Transfer RNA
rRNA: Ribosomal RNA
